# Pesticide residues in water, sediment and fish from Tono Reservoir and their health risk implications

**DOI:** 10.1186/s40064-016-3544-z

**Published:** 2016-10-22

**Authors:** Osei Akoto, Augustine Asore Azuure, K. D. Adotey

**Affiliations:** 1 Department of Chemistry, Kwame Nkrumah University of Science and Technology, Kumasi, Ghana; 2 Department of Theoretical and Applied Biology, Kwame Nkrumah University of Science and Technology, Kumasi, Ghana; 3 Nuclear Chemistry and Environmental Research Center of GAEC, Accra, Ghana

**Keywords:** Bioaccumulation, Fish, Health risk, Pesticides, Toxicity

## Abstract

Levels of organochlorine (OC) and organophosphorus (OP) pesticide residues in fish, sediments and water and their health risk associated with the consumption of the fish from the Tono Reservoir, Ghana were evaluated. The analytical methods included solvent extraction of the pesticide residues using ultrasound sonication and soxhlet extraction and their subsequent quantification using GC equipped with electron capture detector and pulse flame photometric detector after clean-up on activated silica gel/anhydrous sodium sulphate. A total of 29 pesticides comprising 16 OCs and 13 OPs were analyzed, out of which aldrin, *p*,*p*′-DDE and *p*,*p*′-DDD were detected in fish and sediment samples. The results showed that all the residues in water had their concentrations below the detection limit. Mean concentrations of organochlorine pesticide (OCP) residues in fish ranged from 0.017 to 0.17, 0.043 to 0.30, 0.027 to 0.243 and 0.097 to 0.263 µg/g in *Sarotherodon galilaeus*, *Clarias anguillaris*, *Schilbe intermedius* and *Marcusenius senegalensis* respectively. Mean concentrations of organophosphates pesticides ranged from 0.080 to 0.090, 0.080 to 0.087 and 0.050 to 0.063 µg/g in *C. anguillaris*, *S. intermedius* and *M. senegalensis* respectively. The level of chlorpyrifos in *S. galilaeus* was 0.160 µg/g. Mean concentrations of OCP residue in sediments ranged from 0.047 to 0.090 µg/g. Aldrin recorded the highest level while *p*,*p*′-DDD recorded the lowest level. The mean concentrations for all the detected residues were below the WHO/FAO maximum residue limits. Health risk estimation revealed that aldrin in *M. senegalensis* had great potential for systemic toxicity to consumers.

## Background

Reduction of the proliferation of pest and increase in food production, has made pesticides application in agriculture inevitable (Akoto et al. 2013). Pesticides constitute one of the most hazardous groups of contaminants (Vega et al. 2005), posing potential risk to humans and other life forms (Jeyakumar et al. 2014). Thus deaths and chronic diseases worldwide are sometimes reported to have resulted from pesticide poisoning (Rigotto et al. 2013).

The occurrence of pesticides residue, especially organochlorines (OCs) in the environment is a great worry due to their tendency for long-range transport. Also their capacity to bioaccumulate in food chain poses a threat to human health and the environment (Chau 2005; Zhou et al. 2006; Pandit et al. 2006; Guo et al. 2008). Pesticides enter and pollute any component of the environment in a number of ways, including application, accidental spillage or through the unauthorized dumping of pesticide products or their containers (Cox 2002). Contamination of water bodies for example is a major concern for fish and other aquatic organisms such as mussels, oysters, prawns and lobsters which are major sources of protein (Essumang and Chokky 2009). Accumulation of pesticides in these organisms has become a serious public health issue worldwide. Fish are used extensively for environmental monitoring because they concentrate pollutants directly from water and diet, thus enabling the assessment of transfer of pollutants through the food web (Bruggeman 1982; Fisk et al. 1998; Lanfranchi et al. 2006; Das et al. 2002).

Fish occupy different habitats in the ecosystem and have different feeding behaviors, thereby exhibiting different profile of accumulation of contaminants such as pesticides. For example, benthic fish species are considered more prone to contamination (Yim et al. 2005; Wei et al. 2014), as they tend to accumulate sediments bound contaminants than pelagic fish (Qadir and Malik 2011; Ccanccapa et al. 2016).

Although the use of OCs and many types of OPs pesticides has been banned or severely limited in Ghana, they are still being used in many parts of the country because they are effective for agricultural and relatively inexpensive as compared to the cost of other class of pesticides (Ntow et al. 2006; Racke et al. 1997).

The Tono irrigation site near Navrongo in the Republic of Ghana, is noted for the production of large quantities of vegetables. In other to improve yield, there has been a widespread and unguided application of pesticides within the catchment by farmers. This may have resulted in runoff of pesticide residues into the reservoir thereby polluting the ecosystem and contaminating organisms living in the reservoir. It is for this reason that this study was carried out to assess the level of OC and OP residue in fish, water and sediment from the Tono Reservoir and to evaluate the potential health risk posed to consumers by these fish species.

## Methods

### Description of study area

**Fig. 1:**
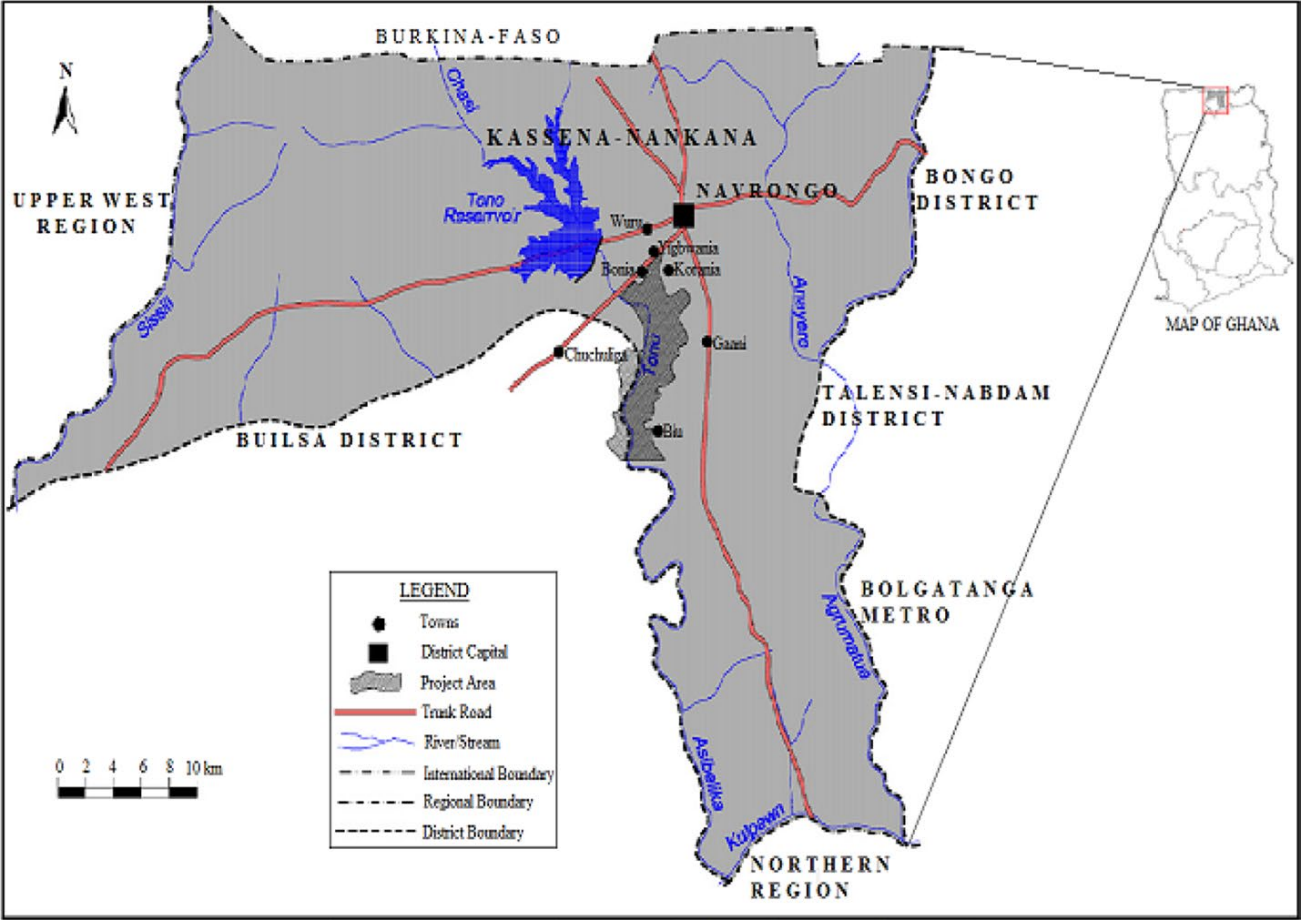
Map of Kassena Nankana district in Northing Ghana showing the Tono Reservoir

The Tono Reservoir is located near the Tono Irrigation Project at Navrongo in Northern Ghana (Fig. 1). This project was established to promote the production of food crops by small-scale farmers within organized and managed irrigation schemes and covers an area of about 2490 ha (Gordon 2006; Ntow 2008). The cropping areas are divided between upland and lowland areas. Crops grown in upland plots include onions, tomatoes, millet, groundnuts, sorghum and maize while the lowland areas are earmarked for rice production. Predominant pesticides used in the area are endosulfan and chlorpyrifos which are supervised and regulated by the irrigation authorities (Ntow 2008). Some farmers also obtain cheap but effective pesticides from neighboring countries such as Togo and Burkina Faso which are not approved by the by EPA, Ghana, the pesticides regulating authority (Okoffo et al. 2016). The dam has a maximum storage capacity of about 5.0 × 10^5^ m^3^ (Pelig-Ba 2011). Fishing in the reservoir is restricted officially and is only allowed on a limited scale.

### Sampling

Water samples were collected from different locations within the reservoir at depth of about 1 m into previously cleaned 2.5 L plastic bottles and covered with screwed caps. A total of 12 water samples were collected from the reservoir. Sediment samples were grabbed from the bottom of the reservoir at the same locations where water samples were collected. The sediment samples were stored in clean polyethylene bags, labelled and sealed. Fish samples were purchased from fishermen at one of the reservoir’s landing site. The samples were wrapped in aluminum foil, packed in clean polyethylene bags, labelled and sealed, and then transported in a thermo-insulated container with ice packs to the laboratories of Nuclear Chemistry and Environmental Research Center of the Ghana Atomic Energy Commission where they were stored at a temperature of −20 °C until processing and analysis. All glassware and containers were washed with detergent, rinsed with purified water and acetone and kept in an oven at 180 °C for 2 h. Sampling was conducted in two batches (August 2014 and March 2015).

Fish samples were identified at the Inland Fisheries Research Division of the Ministry of Food and Agriculture. Fish selected for this study were Catfish (*Clarias anguillaris*), Silver catfish (*Schilbe intermedius*), Trunkfish (*Marcusenius senegalensis*) and Mango Tilapia (*Sarotherodon galilaeus*). Species are among the commonly consumed fishes from the reservoir. A total of 40 fish samples comprising 10 of each species were used for this work.

### Sample preparation and extraction

Water samples were filtered through 0.45 μm fiber glass filters (Whatman) to remove suspended materials. Sediment samples were air dried and then sieved through a 250 µm stainless steel mechanical shaker. Fish samples were thawed, cleaned with distilled water and scales sloughed off. Muscle tissues were dissected, minced into smaller pieces and homogenized.

Extraction of pesticide residues in fish, water and sediment samples was done according to the method developed by Therdteppitak and Yammang (2002) with some modifications.

Ten (10.0) g of homogenized fish sample was placed into a 100 mL conical flask and 20.0 g of activated anhydrous sodium sulfate were added and mixed. Then 30 mL of 2:1 (v/v) hexane/acetone mixture were added and thoroughly mixed by shaking. The sample was then sonicated for 20 min using Bransonic Ultrasound Sonicator. The supernatant was filtered into a 250 mL round bottom flask. The extraction was repeated two more times and all the supernatants combined and concentrated at 40 °C to near dryness using a Vacuum Rotary Evaporator (Buchi rotavapor R-200, Buchi heating bath B-490).

### Extraction of pesticide residues in water samples

About 50 mL *n*-hexane was introduced into a 1 L separating funnel containing 100 mL of filtered water sample. The mixture was shaken vigorously for 5 min and allowed to settle. After complete separation, the organic phase was drained into a 250 mL conical flask while the aqueous phase was re-extracted twice with 50 mL of *n*-hexane. The extracted organic phase was combined and dried by passing through a glass funnel packed with activated anhydrous sodium sulfate. The organic fraction was then concentrated to near dryness using vacuum rotary evaporator at 40 °C.

### Extraction of pesticide residues in sediments

A 10.0 g dry sediment sample was transferred into an extraction thimble that was previously washed with *n*-hexane and acetone and oven dried. Pesticide residues in sediments were extracted with 150 mL of *n*-hexane/acetone mixture 4:1 v/v for 6 h using soxhlet extraction. The extract was then concentrated to near dryness using vacuum rotary evaporator at 40 °C. Each extract was then dissolved in 10 mL of *n*-hexane and subjected to clean-up.

### Sample clean-up

Approximately 2.5 g of activated silica gel was weighed and parked into a glass column which has been plugged with glass wool and 1.0 g of anhydrous sodium sulfate. About 10 mL *n*-hexane was used to wet and rinse the column. The extract was then transferred into the column and eluted with 20 mL portions of hexane/acetone mixtures. The eluates were collected into a round bottomed flask and then concentrated to dryness. The residues were then dissolved in 2 mL of ethyl acetate and placed in a GC vial for gas chromatograph analysis.

### GC analysis

A Shimadzu 2010 GC equipped with an ECD was used to analysed the OCP residues. Separation was done on an SGE BPX-5 of 60 m capillary column with 0.25 mm internal diameter and 0.25 µm film thicknesses, equipped with 1 m retention gap. The oven temperature was programmed as follows: initial temperature was set at 90 °C for 3 min and ramped at 30 °C/min to 200 °C for 15 min and then to 265 °C at a rate of 5 °C/min for 5 min then to 275 °C at the rate of 3 °C/min and allowed to stay for 15 min. The injector setting is a pulsed splitless mode with a temperature of 250 °C at a pressure of 1.441 bar. Pulsed pressure was 4.5 bar, pulsed time 1.5 min, purge flow of 55.4 mL/min with a purge time of 1.4 min. The detector temperature was 300 °C. Nitrogen was used as carrier gas at a flow rate of 30 mL/min.

A Varian CP-3800 GC equipped with a Combi PAL Auto sampler was used to measure levels of the OP residues. The column used was a 30 m × 0.25 mm internal diameter fused silica capillary coated with VF-1701 (0.25 µm film). The oven temperature was programmed as follows: initial temperature was set at 70 °C for 2 min and ramped at 25 °C/min to 200 °C for 6 min and then to 250 °C at 20 °C/min and allowed to stay for 19 min. The injector setting is a pulsed splitless mode at a temperature of 270 °C. The detector temperature was 280 °C in “constant makeup flow” mode. Nitrogen gas was used as carrier gas at a flow rate of 2 mL/min.

### Quality assurance and quality control

Quality control measures were assured through analysis of solvent blanks, procedure blanks. All reagents used were analytical grade. Each sample was analyzed in triplicates and mean concentrations were calculated based on the number of samples that tested positive. Recalibration curves were run with each batch of samples to check their correlation coefficient which was kept above 0.98. The results of assurance analysis indicated that pesticide determinations were within accepted levels of accuracy.

### Estimation of daily intake

The estimated daily intakes (EDIs) of the various pesticides in each fish species was determined by using equation;

$$EDI = \frac{C \times D}{B}$$

where C, D, and B represent the concentration of pesticide residues in fish (µg/g) on wet weight basis, average daily intake of fish estimated at 68.5 g/person/day for adults (Global Fish Alliance 2010) and average body weight considered to be 60 kg for adults (IPCS 2006; Ge 1992).

Health risk assessment of consumers from the intake of pesticides contaminated fish was characterized by using health risk index (HI). The estimated HIs were obtained by dividing the EDI by their corresponding values of acceptable daily intakes (ADI) by WHO/FAO (FAO/WHO 2010) as shown by the equation;

$$HR = \frac{EDI}{ADI}$$

When the HI is less than 1, the food concerned is considered acceptable. If it is greater than 1, the food concerned is considered a risk to the consumer (Darko and Akoto 2008; Akoto et al. 2015).

## Results and discussion

A total of 29 pesticide residues were analyzed. It comprised 16 OCs (β-HCH, δ-HCH, lindane, heptachlor, aldrin, dieldrin, endrin, α-endosulphan, β-endosulphan, endosulphan sulphate, methoxychlor, fenvalerate, γ-chlordane, *p*,*p*′-DDE, *p*,*p*′-DDD and *p*,*p*′-DDT) and 13 OPs (methamidophos, enthoprophos, phorate, dimethoate, diazinon, fonofos, pirimiphos-methyl, fenitrothion, malathion, chlorpyrifos, parathion, chlorfenvinphos and profenofos).

Three organochlorine pesticide residues comprising aldrin, *p*,*p*′-DDE and *p*,*p*′-DDD were detected in the fish and sediment samples analyzed. Their mean residual concentrations, ADIs, EDIs and the corresponding health hazard indices for systemic effects associated with the consumption of different fish species are summarised in Table 1.

**Table 1:** Range, mean concentrations, ADIs, EDIs of OCP residues and their HI associated with the consumption of fish from the Tono Reservoir (N = 10)

Species of fish	Pesticide	Range	Mean ± SD	ADI	EDI	HI	HR
*C. anguilareeis*	Aldrin	0.03–0.06	0.043 ± 0.013	0.0001	4.91 × 10^−5^	0.491	No
	*p*,*p*′-DDE	0.20–0.42	0.310 ± 0.090	0.0020	3.54 × 10^−4^	0.177	No
	*p*,*p*′-DDD	0.14–0.15	0.143 ± 0.005	0.0020	1.63 × 10^−4^	0.082	No
S. galilaeus	Aldrin	0.01–0.03	0.017 ± 0.012	0.0001	1.94 × 10^−5^	0.194	No
	*p*,*p*′-DDE	0.17–0.17	0.170 ± 0.000	0.0020	1.94 × 10^−4^	0.097	No
	*p*,*p*′-DDD	0.02–0.18	0.083 ± 0.085	0.0020	9.48 × 10^−5^	0.047	No
*S. intermedius*	Aldrin	0.02–0.04	0.027 ± 0.012	0.0001	3.08 × 10^−5^	0.308	No
	*p*,*p*′-DDE	0.19–0.34	0.243 ± 0.067	0.0020	2.77 × 10^−4^	0.139	No
	*p*,*p*′-DDD	0.02–0.14	0.093 ± 0.064	0.0020	1.06 × 10^−4^	0.053	No
*M. senegalensis*	Aldrin	0.01–0.15	0.097 ± 0.078	0.0001	1.11 × 10^−4^	1.107	Yes
	*p*,*p*′-DDE	0.22–0.30	0.263 ± 0.033	0.0020	3.00 × 10^−4^	0.150	No
	*p*,*p*′-DDD	0.08–0.16	0.110 ± 0.044	0.0020	1.26 × 10^−4^	0.063	No

The mean concentration of the OCPs detected in *S. galilaeus* samples were 0.017 ± 0.012, 0.170 ± 0.001 and 0.083 ± 0.085 µg/g for aldrin, *p*,*p*′-DDDE and *p*,*p*′-DDD, respectively. *p*,*p*′-DDE which recorded the highest mean concentration in *S. galilaeus* was detected in two samples. The least mean concentration in this species was recorded by aldrin which was detected in 30 % of the samples. The mean concentrations of aldrin, *p*,*p*′-DDE and *p*,*p*′-DDD in *S. galilaeus* were all below the FAO/WHO (2009) maximum residue limits (MRLs) of 0.2, 1.0 and 1.0 µg/g respectively.

The mean concentrations of the three OCs that were detected in *C. anguilaris* samples were 0.043 ± 0.013, 0.310 ± 0.090 and 0.143 ± 0.005 µg/g for aldrin, *p*,*p*′-DDDE and *p*,*p*′-DDD, respectively (Table 1). *p*,*p*′-DDE which was detected in four samples, recorded the highest mean concentration in *C. anguilaris*. Aldrin was detected in four samples and had the lowest mean concentration. All mean concentrations of OCPs in *C. anguilaris* were below the FAO/WHO (2009) MRLs.

OCP residues detected in *S. intermedius* samples were aldrin, *p*,*p*′-DDE and *p*,*p*′-DDD at concentration 0.027 ± 0.012, 0.243 ± 0.067 and 0.093 ± 0.064 µg/g respectively (Table 1). *p*,*p*′-DDE, the most frequent residue with the highest mean concentrations of 0.243 ± 0.067 µg/g occurred in 4 samples of the *S. intermedius*. The minimum residue level of 0.027 ± 0.012 µg/g was recorded by aldrin detected in 3 samples.

Organochlorine pesticide residues detected in *M. senegalensis* were aldrin, *p*,*p*′-DDE and *p*,*p*′-DDD as presented in Table 1. *p*,*p*′-DDE recorded the highest mean concentration of 0.263 ± 0.033 µg/g and occurred in 4 of the samples. The mean concentrations of OCPs in *M. senegalensis* did not exceed the FAO/WHO (2009) MRLs.

Higher levels of DDT metabolites; *p*,*p*′-DDD and *p*,*p*′-DDE were recorded in the four species of fish samples. These results indicate that *p*,*p*′-DDE accumulation was the highest in all the fish samples. Its highest value was observed in *C. anguilaris* at a level of 0.310 ± 0.090 µg/g, while the lowest mean value of 0.170 ± 0.000 µg/g was observed in *S. galilaeus*. In a similar study, Zabik et al. (1995) found that over 80 % of DDTs detected in fish samples from great lakes were *p*,*p*′-DDE. The current study also agree with Kuranchie-Mensah et al. (2011) who found widespread of *p*,*p*′-DDE in all fish samples from the Volta Lake in Ghana. The presence of *p*,*p*′-DDE in muscle tissues of fish has also been reported from Lake Parishan in Iran by Kafilzadeh et al. (2012). The results from this study is expected because of the high lipophilic and hydrophobic nature of DDT and its metabolites (*p*,*p*′-DDD and *p*,*p*′-DDE) and the possibility of being retained on the organic phase of sediment and organisms as indicated by Adeyemi et al. (2008). Again, the detection of *p*,*p*′-DDE and *p*,*p*′-DDD is an indication of photochemical degradation of *p*,*p*′-DDT which shows past use of *p*,*p*′-DDT within the Tono catchment. Thus farmers within the Tono irrigation site have probably stopped using DDT on their farms.

Aldrin was detected in all the species of fish analyzed. The maximum mean concentration of aldrin occurred in *M. senegalensis* while the minimum level was found in *S. galilaeus*. Research has shown that aldrin is readily converted into dieldrin in plant and animal tissues and also, aldrin photolysis to dieldrin in the environment (Akan et al. 2014). It can therefore be stated that the presence of aldrin without it’s metabolite in this study was an indication of recent use of aldrin within the Tono catchment even though the use of this pesticide is completely banned in Ghana.

*Clarias anguillaris* recorded the highest total mean residues of the OCPs followed by *M. senegalensis* and *S. intermedius* whilst the least was recorded in *S. galilaeus*. The differences in concentration in the fishes are probably due to different feeding habits. Carnivorous fish might bioaccumulate OC pesticides more by eating other fishes, while the constant contact of bottom feeders like *M. senegalensis* with sediments allows their continuous exposure to the adsorbed pesticides. *C. anguillaris* and *S. intermedius* are carnivorous fishes which are high in the trophic level and are thus potentially prone to biomagnification as compared to *S. galilaeus* (Kuranchie-Mensah et al. 2011) which feeds primarily on phytoplankton and algae. In a study by (Kent and Johnson 1979) *C. anguilaris* which is a bottom feeder, recorded the highest level of OC pesticides in the American Fall Reservoir. Again, data from the National Contaminant Biomonitoring Program in major US Rivers and the Great lakes found no difference between OC pesticide residues in bottom feeders and predatory fish (Caldas et al. 1999). A similar observation was also recorded in this study, where the benthic feeders *M. senegalensis* and predator *S. intermedius* contained higher concentrations of total OC residues than the herbivore *S. galilaeus*. Studies have shown that species-specific differences and feeding habit of fishes also influence the variation in accumulation pattern of OCP residues among species (Muralidharan et al. 2009).

### OP pesticides residue levels in fish samples from Tono Reservoir

Mean residual concentrations, EDI and health risk estimation for OPs in the fish samples from the Tono Reservoir are presented in Table 2. Out of the thirteen (13) OPs analysed only chlorpyrifos and pirimiphos-methyl were detected in fish samples. Mean concentrations of chlorpyrifos in muscle tissues of *C. anguilaris* was 0.093 ± 0.074 µg/g while a mean concentration of 0.080 ± 0.066 µg/g was recorded for pirimiphos-methyl in 50 % of the samples. Chlorpyrifos and pirimiphos-methyl residues were detected in *S. intermedius* and *M. senegalensis* samples. Chlorpyrifos recorded mean concentrations of 0.087 ± 0.038 and 0.050 ± 0.057 µg/g in *S. intermedius* and *M. senegalensis* respectively. Pirimiphos-methyl recorded a mean concentration of 0.080 ± 0.046 µg/g in *S. intermedius* and a mean concentration of 0.063 ± 0.045 µg/g in *M. senegalensis.* Chlorpyrifos was the only residue found in S*. galilaeus* samples from the Tono Reservoir at a mean level of 0.160 ± 0.037 µg/g. This was detected in 40 % of the total samples.

**Table 2:** Range, mean concentrations, ADIs, EDIs of OP pesticides residues and their HI associated with the consumption of fish from the Tono Reservoir (N = 10)

Species of fish	Pesticide	Range	Mean ± SD	ADI	EADI	HI	HR
*C. anguillaris*	Chlorpyrifos	0.01–0.15	0.093 ± 0.074	0.0100	1.06 × 10^−4^	0.011	No
	Pirimiphos-methyl	0.01–0.15	0.080 ± 0.066	0.0200	9.13 × 10^−5^	0.005	No
*S. intermedius*	Chlorpyrifos	0.06–0.13	0.087 ± 0.038	0.0100	9.93 × 10^−5^	0.010	No
	Pirimiphos-methyl	0.04–0.13	0.080 ± 0.046	0.0200	9.13 × 10^−5^	0.005	No
*M. senegalensis*	Chlorpyrifos	0.01–0.13	0.050 ± 0.057	0.0100	5.71 × 10^−5^	0.006	No
	Pirimiphos-methyl	0.02–0.11	0.063 ± 0.045	0.0200	7.19 × 10^−5^	0.004	No
*S. galilaeuls*	Chlorpyrifos	0.11–0.20	0.160 ± 0.037	0.0100	1.83 × 10^−4^	0.018	No

Organophosphorus pesticides are much more resistant to microbial degradation and have the tendency to concentrate in lipid rich tissues of aquatic organisms (Essumang and Chokky 2009). Among the two OPs detected, chlorpyrifos recorded the highest concentration in *S. galilaeus* while the least value was determined in *M*. *senegalensis.* Pirimiphos-methyl was however not detected in all the samples of *S. galilaeus*. The highest mean total OP residual load was recorded in *C. anguillaris* followed by *S. intermedius* and *S. galilaeus* while the lowest value was recorded in *M. senegalensis*. The presence of the detected OP pesticides residues is evident by their use on irrigation farms around the Tono Reservoir. Organophosphorus pesticide residues were below the prescribed MRL set by FAO/WHO in all fish samples. Similar work conducted in Nigeria by Akan et al. (2014) reported significantly higher concentrations of chlorpyrifos (ranging from 0.77 to 2.22 µg/g) in fish tissues than those detected in this study.

### OC pesticide residues in sediments from Tono Reservoir

Levels of OCP residues detected in surface sediment samples from Tono Reservoir are presented in Table 3. Out of 16 organochlorines analyzed, residues of three (aldrin, *p*,*p*′-DDE and *p*,*p*′-DDD) were detected in sediment samples with aldrin recording the highest mean residue level of 0.090 ± 0.050 µg/g followed by *p*,*p*′-DDE which recorded a mean concentration of 0.070 ± 0.033 µg/g. *p*,*p*′-DDD recorded the lowest mean concentration of 0.047 ± 0.028 µg/g.

**Table 3:** Levels of OC and OP pesticides residues in the sediments from the Tono Reservoir

Class of pesticide	Pesticide	Range	Mean ± SD
Organochlorines	Aldrin	0.04–0.14	0.090 ± 0.050
	*p*,*p*′-DDE	0.01–0.11	0.070 ± 0.033
	*p*,*p*′-DDD	0.02–0.09	0.047 ± 0.028
Organophosphorus	Pirimiphos-methyl	0.01–0.17	0.104 ± 0.052
	Profenofos	0.01–0.15	0.050 ± 0.047

Studies have shown that OC pesticides tend to accumulate more in aquatic organisms and they substantially settle on the sediments (Akan et al. 2014). The result of this study indicates that OC pesticide residues were present in varying amounts in different species of fish and sediments sampled from Tono Reservoir. Indeed, the study has revealed that any OC pesticide present in water will preferably be adsorbed to sediment or bioaccumulate in fish due to low water solubility of OC pesticides (Caldas et al. 1999). This could probably be the reason for the absence of OCP in water samples analyzed in this study. On the contrary, work done by Pelig-Ba (2011) on unfiltered water from the Tono Reservoir revealed presence of aldrin (0.002 µg/L), p,p-DDE (0.002 µg/L) and DDT (22.4 µg/L).

The mean concentrations of *p*,*p*′-DDE and *p*,*p*′-DDD determined in fish samples were higher than those present in sediments. Sediments however had higher concentrations of aldrin than fish samples except in M*. senegalensis*. Sediment samples also had more frequent detection of pesticides than fish samples. This contrast to findings made by Caldas et al. (1999) in which fish samples from Paranoa Lake of Brasilia in Brazil had more frequent detections of OCP residue than did sediment samples from the same lake. Kuranchie-Mensah et al. (2011) reported much higher mean concentrations of aldrin and *p*,*p*′-DDE in sediments at Nsawam in Ghana from the Densu river basin than the levels determined in this study.

### OP pesticide residues in sediments from Tono Reservoir

In the sediment samples, two OP pesticides residues (pirimiphos-methyl and profenofos) were detected. Pirimiphos-methyl recorded the highest mean residual value while profenofos recorded the lowest (Table 3). Pirimiphos-methyl was found in both sediments and all the fish samples analyzed except in *S. galilaeus*. The levels of pirimiphos-methyl in sediments were higher than the levels in the fish samples. Profenofos on the other hand was detected only in sediments. Even though chlorpyrifos was detected in the fish samples, there was no detection of chlorpyrifos in sediment samples.

None of the two pesticides classes considered in this study was detected in the water samples. These pesticides are lipophilic and therefore insoluble in water.

### Potential health risk associated with the consumption of fish contaminated with OCP and OPP residues from the Tono Reservoir

The hazard indices presented in Table 1 showed that aldrin in *M. senegalensis* recorded HI > 1. This shows that there is health risk associated with lifetime consumption of *M. senegalensis* from the Tono Reservoir. On the other hand, *S. galilaeus, C.anguillaris and S. intermedius* showed no health hazard associated with their consumption as their hazard indices for all the detected residues (aldrin, *p*,*p*′-DDE and *p*,*p*′-DDD) were <1 in spite of the presence of OCP and OPP residue in the fish.

## Conclusion

The study has revealed the presence of aldrin, *p*,*p*′-DDE, *p*,*p*′-DDD, chlorpyrifos, pirimiphos-methyl and profenofos in fish and sediment from the Tono Reservoir but all pesticides were below detection limits in the filtered water. Concentrations of the OCP residues detected in the fish samples were higher than that in the sediments. The levels of all the pesticide residues found in this study were well below WHO/FAO values. Analysis of health risk assessment revealed that aldrin in *M. senegalensis* had great potential for systemic toxicity to consumers. Regular monitoring is therefore required to control the levels of pesticide residues in the reservoir.
